# Lifestyle factors related to prevalent chronic disease multimorbidity: A population-based cross-sectional study

**DOI:** 10.1371/journal.pone.0287263

**Published:** 2023-07-24

**Authors:** Jacobien Niebuur, Judith M. Vonk, Yihui Du, Geertruida H. de Bock, Gerton Lunter, Paul F. M. Krabbe, Behrooz Z. Alizadeh, Harold Snieder, Nynke Smidt, Marike Boezen, Eva Corpeleijn

**Affiliations:** Department of Epidemiology, University of Groningen, University Medical Center Groningen, Groningen, The Netherlands; Taipei Medical University, TAIWAN

## Abstract

**Background:**

Multimorbidity is associated with poor quality of life, polypharmacy, health care costs and mortality, with those affected potentially benefitting from a healthy lifestyle. We assessed a comprehensive set of lifestyle factors in relation to multimorbidity with major chronic diseases.

**Methods:**

This cross-sectional study utilised baseline data for adults from the prospective Lifelines Cohort in the north of the Netherlands (N = 79,345). We defined multimorbidity as the co-existence of two or more chronic diseases (i.e. cardiovascular disease, cancer, respiratory disease, type 2 diabetes) and evaluated factors in six lifestyle domains (nutrition, physical (in)activity, substance abuse, sleep, stress, relationships) among groups by the number of chronic diseases (≥2, 1, 0). Multinomial logistic regression models were created, adjusted for appropriate confounders, and odds ratios (OR) with 95% confidence intervals (95%CI) were reported.

**Results:**

3,712 participants had multimorbidity (4.7%, age 53.5 ± 12.5 years), and this group tended to have less healthy lifestyles. Compared to those without chronic diseases, those with multimorbidity reported physical inactivity more often (OR, 1.15; 95%CI, 1.06–1.25; not significant for one condition), chronic stress (OR, 2.14; 95%CI, 1.92–2.38) and inadequate sleep (OR, 1.70; 95%CI, 1.41–2.06); as expected, they more often watched television (OR, 1.70; 95%CI, 1.42–2.04) and currently smoked (OR, 1.91; 95%CI, 1.73–2.11), but they also had lower alcohol intakes (OR, 0.66; 95%CI, 0.59–0.74).

**Conclusions:**

Chronic stress and poor sleep, in addition to physical inactivity and smoking, are lifestyle factors of great concern in patients with multimorbidity.

## Introduction

Multimorbidity, defined as the co-existence of two or more chronic diseases in the same individual, is a global health priority [1]. It typically includes diseases from among the ‘Big Four,’ namely cardiovascular disease (CVD), cancer, chronic respiratory diseases and type 2 diabetes (T2D), which together account for 57% of global mortality [2]. In addition to high mortality rates, disability, functional decline and poor quality of life [1, 3], multimorbidity places a substantial economic burden on society due to exponentially rising healthcare costs [4]. Prevalence estimates for multimorbidity vary from 13% (age ≥18 years) to 95% (age ≥85 years) depending on the definition used, the diseases included, and the population studied [3, 5–7]. However, the prevalence is increasing rapidly as populations age, putting ever greater pressures on healthcare systems [4, 8]. The Big Four share modifiable lifestyle risk factors, including physical inactivity, unhealthy diet, high alcohol consumption and smoking, as targeted by the World Health Organization (WHO) to lower the burden of chronic disease [9]. The new field of lifestyle medicine adds restorative sleep, adequate psychological stress management and social connectedness as related factors [10, 11].

The recognition that lifestyle interventions can improve clinical care has given rise to the field of lifestyle medicine. This discipline can benefit patients with more complex needs because limitations in daily function and polypharmacy have their greatest affects in this group. Lifestyle interventions can help alleviate complaints [12], support medication effectiveness [13] and support disease remission [14], all while avoiding further medication prescriptions.

Contrasting with individual chronic diseases, we know relatively little about the associations of lifestyle factors with multimorbidity [15]. Research has found associations between some unhealthy behaviours and prevalent cardiometabolic multimorbidity [16], prevalent chronic disease multimorbidity [17, 18], incident cancer and cardiometabolic disease multimorbidity [15], and incident chronic disease multimorbidity [19]. However, those studies evaluated a limited number of lifestyle factors (smoking, alcohol consumption, physical inactivity or diet quality) and factors like sleep, stress and social connectedness were not included. In addition, a large variation exists in the definitions of multimorbidity, making the conclusions not comparable. Understanding the extent to which lifestyle-related risk factors are present and specific for multimorbidity related to the Big Four chronic diseases will help uncover future directions for clinical care.

We aimed to evaluate the cross-sectional association between relevant lifestyle domains and multimorbidity. Relevant lifestyle domains included nutrition, exercise, stress, substance abuse, sleep and relationships, while multimorbidity indicated two or more from among CVD, cancer, respiratory disease and T2D.

## Materials and methods

### Study population and design

This cross-sectional study utilised the rich contemporary data from the Lifelines Cohort Study [20] to assess the associations between a comprehensive set of lifestyle factors and multimorbidity. Lifelines is a multi-disciplinary, prospective, population-based cohort study examining the health and health-related behaviours (focusing on multimorbidity) of 167,729 persons living in the north of the Netherlands. Full details of the cohort are described elsewhere [20]. The Lifelines Cohort Study was approved by the medical ethics committee of the University Medical Center Groningen, the Netherlands. Written consent was obtained from all participants.

The present study used baseline data for adults (age ≥18 years), collected between December 2006 and December 2013, if it included all lifestyle factors and all four outcome measures needed to calculate multimorbidity.

### Outcome measures

The main outcome was multimorbidity. Definition and measurement of multimorbidity is highly variable in the literature [21]. We focused on the four major non-communicable diseases of CVD, cancer, chronic respiratory disease and T2D because of their clinical relevance for healthy ageing. Multimorbidity was defined as the co-existence of at least two diseases from those four. According to Wister et al [22], additive multimorbidity scale is a better measure of multimorbidity than dichotomous measures. Therefore, we grouped the participants based on the count of chronic diseases. However, to ensure a sufficient sample size in each group, participants with 2, 3 or 4 chronic diseases were grouped together. ‘No disease’ was defined as having none, and ‘single disease’ as having one of the four major chronic diseases.

#### Cardiovascular diseases

We defined 11 CVDs at baseline [23] based on the ICD-10, including myocardial infarction, heart failure, atrial fibrillation, heart valve disorders, arrhythmia, aortic aneurysm, stroke, thrombosis, atherosclerosis, narrowing carotid arteries and balloon angioplasty or bypass surgery [24]. The operationalization methods developed by Van der Ende et al. [23] to validate myocardial infarction, heart failure and atrial fibrillation with medication or ECG abnormalities were used, and the other CVDs relied on self-reported information. Participants were considered prevalent cases if they suffered from at least one of these CVDs.

#### Cancers

The cancer assessment relied on self-reported information. When assessing chronicity, we excluded participants with primary skin cancer, pre-stage cancer, or past cancer with an unknown primary location from the definition of prevalent cancer. This left four clusters of chronic cancer: solid (including breast cancer and endometrial cancer), bone and soft tissue, hematological (including lymph and leukemia), and central nervous system.

#### Chronic respiratory diseases

We defined two phenotypes when assessing chronic respiratory disease: airflow obstruction and asthma. Airflow obstruction, assessed by pre-bronchodilator spirometry (measured using a Welch Allyn Version 1.6.0.489, PC-based SpiroPerfect with Ca Workstation software), was defined as a ratio between the forced expiratory volume in one second and the forced vital capacity (FEV_1_/FVC) below the lower limit of normal (LLN) [25]. The LLN reference value was based on age, gender, height and ethnicity, and was calculated as the lower fifth percentile of a healthy reference population [25, 26]. Asthma was considered present if either a doctor had made the diagnosis or the patient used asthma medication and reported two or more symptoms from among wheeze, dyspnea at rest, and wakened by dyspnea. We considered prevalent cases for chronic respiratory disease those with either airway obstruction, asthma, or both.

#### Type 2 diabetes

Prevalent cases of T2D comprised those (a) with self-reported T2D or reported diabetes with missing or unknown data for the type, and (b) who used diabetes medication and/or had a fasting plasma glucose (FG) ≥7.0 mmol/L and/or a hemoglobin A1c (HbA1c) ≥6.5% [27]. All participants with self-reported diabetes and age of onset < 30 years were not classified as T2D unless they explicitly reported the diagnosis and used antidiabetic medication other than insulin.

### Exposures

We included lifestyle factors from all six domains (nutrition, exercise, stress, substance abuse, sleep, relationships) in the definition of lifestyle medicine [11].

#### Nutrition

Diet quality was assessed using the Lifelines Diet Score (LLDS) [28] and diet was assessed using the food frequency questionnaire (FFQ) over the previous month [29]. The LLDS ranks the relative intake of nine food groups with proven positive health effects and three food groups with proven negative health effects [28]. The positive food groups included vegetables, fruit, whole-grain products, legumes and nuts, fish, oils and soft margarines, unsweetened dairy, coffee, and tea. The negative food groups included red and processed meat, butter and hard margarines, and sugar-sweetened beverages. Scores range from 0 to 48, with higher scores indicating healthier diets. The LLDS was categorized into tertiles.

#### Exercise

The Lifelines Physical Activity Score (LLPAS) [30, 31] was used to assess physical activity, based on the Short Questionnaire to Assess Health-Enhancing Physical Activity [32]. It indicates the minutes moderate-to-vigorous physical activity (MVPA) per week engaged in sports, leisure time and active commuting [30, 31]. We categorized the LLPAS as < 150, 150–300 and ≥300 min of MVPA/week to assess adherence to WHO physical activity guidelines.

The WHO physical activity guidelines also recommend limiting sedentary time. We measured sedentary behavior based on average hours per day watching TV, categorized into ≥5 hours/day, 3–4 hours/day and ≤2 hours/day based on previously reported thresholds for increased risk of negative health outcomes [33].

#### Stress

The Dutch version of the List of Threatening Experiences (LTE) [34] was used to collect information on acute stress. This includes 12 stressful life events in family, work and health domains, for which participants indicated whether it occurred in the past year (yes/no). The total LTE score, which could range from 0 to 12, was categorized as low (0), medium (1) and high (≥2) acute stress.

The Long-term Difficulties Inventory (LDI) [34], which comprises twelve items evaluating the experienced chronic stress in family, work and health domains was used to collect information on chronic stress. For each item, participants indicated how much difficulty and stress they experienced in the last year on a three-point scale (0, not stressful; 1, slightly stressful; 2, very stressful), giving a total LDI in the range 0–24 for categorization as low (0), medium (1–2) and high (≥3) chronic stress considering the sample size distribution of the score.

#### Substance abuse

Alcohol consumption was assessed by dividing the average number of glasses of alcohol per drinking day by the number of drinking days per month from the FFQ data [29]. Binge drinking was defined as consuming ≥4 drinks (females) and ≥5 drinks (males) per drinking day [35]. Consumption was then categorized as high (>1.5 drinks/day on average [36] and/or binge drinking), light-to-moderate (>0 and ≤1.5 drinks/day on average, without binge drinking) and none.

We also grouped participants into current, former and never smoker groups. Current smokers reported smoking in the last month. Former smokers reported having not smoked during the last month, but having smoked for at least one year. Never smokers must not have smoked during the last month and must not have smoked for longer than a year [37].

#### Sleep

Total self-reported sleep per 24 hours was categorised according to the age-specific American National Sleep Foundation recommendations as adequate (7–9 hours if aged 18–64 years and 7–8 hours if ≥65 years), marginal (6 or 10–11 hours if 18–25 years; 6 or 10 hours if 26–64 years; 5–6 or 9 hours if ≥65 years), and inadequate (< 6 or >11 hours if 18–25 years; < 6 or >10 hours if 25–64 years; < 5 or >9 if ≥65 years) [38]. Values ≤2 hours and ≥20 hours were considered implausible, recorded missing [39] and excluded. We also included the self-reported use of sleep medication and tranquillizers during the past year.

#### Relationships

Social connectedness indicated the number of contacts (i.e. the average number of people with whom the participant had regular contact about personal aspects) over a two-weeks period, and was categorised by tertile as low (≤9), medium (10–19) and high (≥20).

Family support was defined by marital status and categorised as no partner, relationship without cohabiting, and relationship with cohabiting, including being married.

### Covariates

Based on the social determinants of health in patients with multimorbidity, we included several covariates [40]. Socioeconomic status was measured by questions about educational attainment and household equivalent income. Educational attainment was categorised as elementary (no education or primary education), lower secondary (lower or preparatory vocational education or lower general secondary education), upper secondary (intermediate vocational education, apprenticeship, higher general secondary education or pre-university secondary education) or tertiary (higher vocational education or university) [41]. Household equivalent income was calculated by dividing the net household income (the midpoint of each participant’s income category) by the square root of the number of persons living in the household [42] and was grouped into the following categories by income in euros per month: < 1100, 1100–1499, 1500–1899, ≥1900 euro per month, and ‘did not know or did not want to tell’. Finally, body mass index (BMI) was categorised as underweight (BMI < 18.5 kg/m^2^), normal weight (18.5 ≤ BMI< 25.0 kg/m^2^), overweight (25 ≤ BMI < 30.0 kg/m^2^) and obesity (BMI ≥30.0 kg/m^2^).

### Statistical analysis

Descriptive data are presented as means with standard deviations (SD) and ranges, or as percentages. We used multinomial logistic regression models to evaluate the association between lifestyle factors and multimorbidity. The nominal outcome variable comprised ‘multimorbidity’, ‘single disease’ and ‘no disease’ categories. To assess the associations between lifestyle factors and multimorbidity compared to having no disease and a single disease, we fitted one model with ‘no disease’ as the reference category and another model with ‘single disease’ as the reference category. All multivariable models were adjusted for age, gender, socioeconomic status [6] and weight status [15, 17, 19], which are established risk factors for multimorbidity, and mutually adjusted for the lifestyle factors. To assess associations between lifestyle factors and each chronic disease separately, we performed binary logistic regression analyses. Odds ratios (ORs) and 95% confidence intervals (95%CIs) are reported. The difference of ORs for multimorbidity vs no disease or vs 1 disease, and for single disease vs no disease was assessed by looking at the non-overlap in ORs with 95%CI. We used IBM SPSS 25 (IBM Corp., Armonk, NY, USA) and considered two-sided *P*-values < 0.05 statistically significant for all analyses.

## Results

### Participant characteristics

Of the 152,728 participants in the Lifelines adult sample at baseline, we excluded 47,179 without information for the Big Four chronic diseases and 26,204 without information for at least one lifestyle domain. This resulted in a final analysis set containing 79,345 participants (**Table 1**). Differences in the statistically significant variables between the included and excluded participants were small in absolute terms (**S1 Table in S1 File**). For example, the analysis set had slightly higher education, household equivalent income, alcohol consumption and cohabitation with a partner.

**Table 1:** Sample characteristics by number of chronic diseases.

		Total	Number of chronic diseases		
			≥2	1	0
Total N (%)		79,345	3,712 (4.7)	22,483 (28.3)	53,150 (67.0)
Mean age (years)		44.4 (12.5)	53.5 (12.5)	45.7 (13.0)	43.1 (11.9)
Age range (min–max, years)		18–90	18–87	18–90	18–88
Male Sex		40.6	40.2	40.9	40.5
Educational Attainment					
	Elementary	2.0	5.4	2.6	1.5
	Lower secondary	25.8	38.7	28.6	23.7
	Upper secondary	40.1	33.0	39.5	40.8
	Tertiary	32.1	22.9	29.2	34.0
Household Equivalent Income (€/month)					
	< 1100	16.6	17.7	17.1	16.2
	1100–1499	22.6	22.1	22.7	22.6
	1500–1899	26.6	22.4	25.6	27.3
	≥1900	20.4	21.5	20.5	20.3
	Don’t know/don’t tell	13.8	16.2	14.0	13.6
BMI (kg/m^2^)		26.0 (4.2)	27.8 (5.0)	26.3 (4.4)	25.7 (4.1)
Weight status					
	Underweight	0.7	0.3	0.7	0.7
	Normal weight	44.4	29.7	41.6	46.7
	Overweight	39.6	42.7	40.4	39.1
	Obesity	15.2	27.3	17.3	13.5

Data are presented as percentages within variable categories, or as mean (SD). Distribution of all variables was significantly different between categories of number of diseases (p< 0.05) except for sex (p = 0.44).

**Fig 1:**
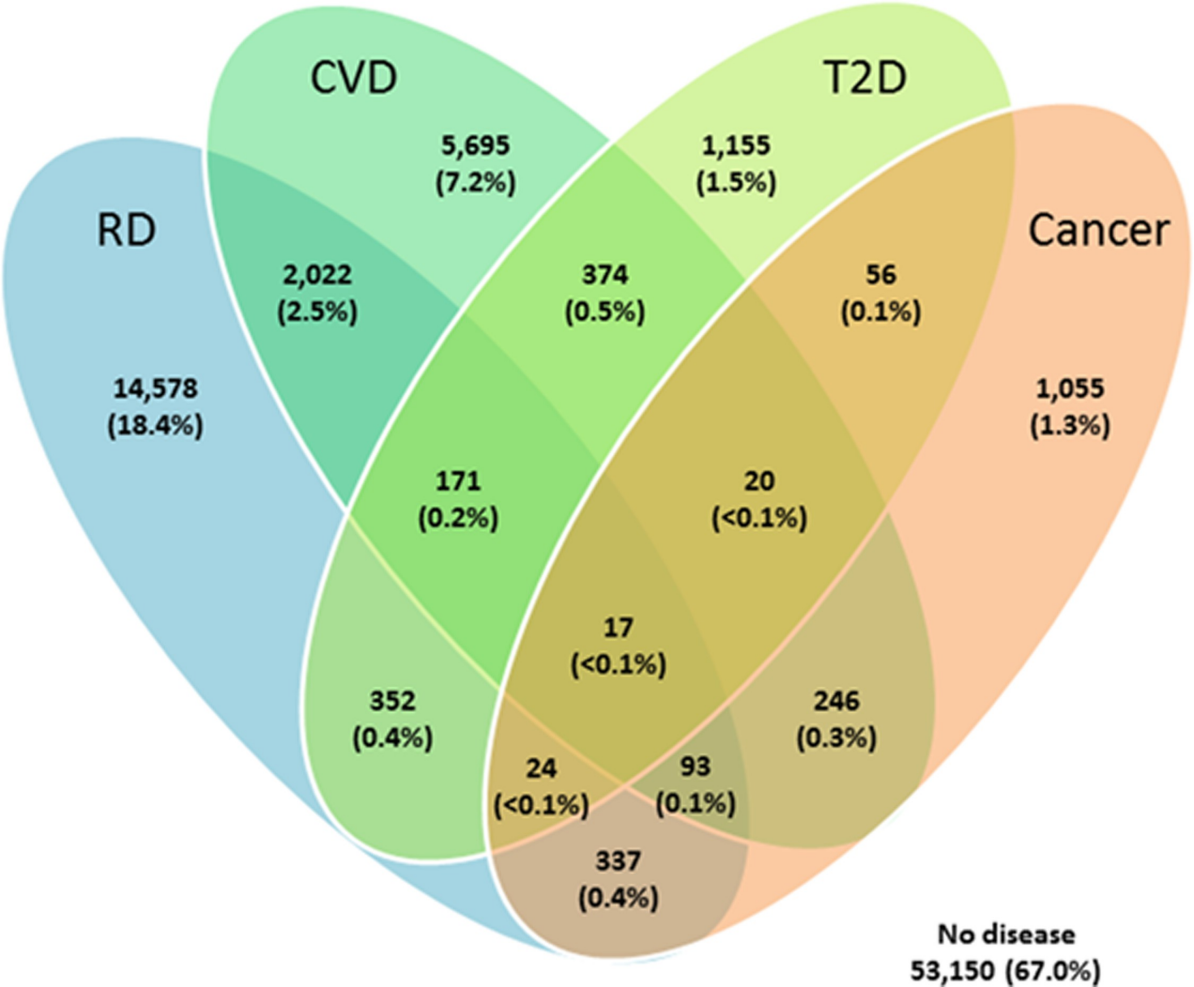
Overlap in the prevalence of The Big Four chronic diseases. The Big Four chronic diseases are respiratory disease, cardiovascular disease, type 2 diabetes and cancer. Data are presented as number (%) from a total of 26,195 participants with at least one chronic disease. The full sample comprised 79,345 participants, of which 53,150 had no major chronic disease. Abbreviations: CVD, cardiovascular disease; RD, Respiratory disease; T2D, Type 2 Diabetes.

Overall, 22,483 (28.3%) had a single chronic disease and 3,712 (4.7%) had multimorbidity, with 3,387 (4.3%) having two chronic diseases. The multimorbidity group, on average, were more often older and overweight or obese compared with the single disease and no disease groups (**Table 1**). Chronic respiratory disease (22.2%) was most common, followed by CVD (10.9%), T2D (2.7%) and cancer (2.3%) (**S1 Table in S1 File)**. Clustering (**Fig 1**) showed that the two most prevalent chronic diseases (CVD and respiratory disease) formed the most prevalent multimorbidity combination.

### Distribution of lifestyle factors by number of chronic diseases

**Table 2** shows the prevalence of the lifestyle factors. Participants with multimorbidity showed lower levels of physical activity, longer TV watching, smoking, inadequate sleep and higher stress, but they reported healthier diets, more alcohol abstinence and more often having quit smoking. They also cohabited with a partner or were single slightly more often than participants with one or no chronic disease, but with no difference in the number of contacts. **S2 Table in S1 File** shows the lifestyle characteristics stratified by each major chronic disease.

**Table 2:** Frequency distributions of lifestyle factors by number of chronic diseases.

Lifestyle factors		Total	Number of chronic diseases		
			≥2 (Multimorbidity)	1 (Single disease)	0 (No disease)
**Number**, n (%)		79,345	3,712 (4.7)	22,483 (28.3)	53,150 (67.0)
**Nutrition**					
Diet quality (LLDS)					
	High (T3, score 27–44)	34.3	38.9	34.4	33.9
	Middle (T2, score 22–26)	37.0	35.9	36.9	37.1
	Low (T1, score 1–21)	28.7	25.2	28.7	28.9
**Exercise**					
Physical activity					
	High (≥300 min/week)	34.7	33.3	34.7	34.7
	Middle (150–299 min/week)	24.7	21.2	23.5	25.4
	Low (< 150 min/week)	40.7	45.5	41.8	39.9
TV watching					
	Low (≤2 hrs/day)	51.5	39.3	48.8	53.4
	Middle (3–4 hrs/day)	46.1	55.5	48.0	44.6
	High (≥5 hrs/day)	2.4	5.2	3.2	1.9
**Stress**					
Stressful life event					
	Low (0 events)	44.2	35.5	41.4	45.9
	Middle (1 event)	27.4	27.6	27.6	27.3
	High (≥2 events)	28.5	37.0	31.0	26.8
Chronic stress					
	Low (0 factors)	21.5	21.4	20.8	21.8
	Middle (1 or 2 factors)	37.9	37.7	36.6	38.4
	High (≥3 factors)	40.6	40.9	42.6	39.8
**Substance abuse**					
Alcohol consumption					
	No	16.8	23.7	18.0	15.7
	Light/moderate	61.0	54.2	59.3	62.1
	High	22.3	22.0	22.7	22.1
Smoking habits					
	Never smoker	46.4	31.3	42.3	49.1
	Former smoker	33.0	45.7	34.6	31.5
	Current smoker	20.6	23.0	23.1	19.4
**Sleep**					
Sleep duration					
	Adequate	84.5	77.8	82.6	85.7
	Marginal	13.7	18.1	15.1	12.8
	Inadequate	1.8	4.1	2.3	1.5
Sleep medication use					
	Yes	7.7	9.9	8.3	7.2
**Relationships**					
Number of contacts					
	High (≥20)	35.5	36.2	36.0	35.3
	Middle (10–19)	35.0	34.4	34.3	35.3
	Low (≤9)	29.5	29.5	29.7	29.4
Marital Status					
	Relationship with cohabiting	81.1	81.8	80.5	81.3
	Relationship with no cohabiting	5.2	3.4	5.3	5.3
	No partner	13.7	14.8	14.2	13.4

Data are shown as percentages within disease categories. The frequency distributions of all lifestyle variables were significantly different between categories of number of diseases (p< 0.05) except for ‘number of contacts’ (p = 0.11).

### Association of prevalent single disease and multimorbidity with lifestyle factors

**Fig 2:**
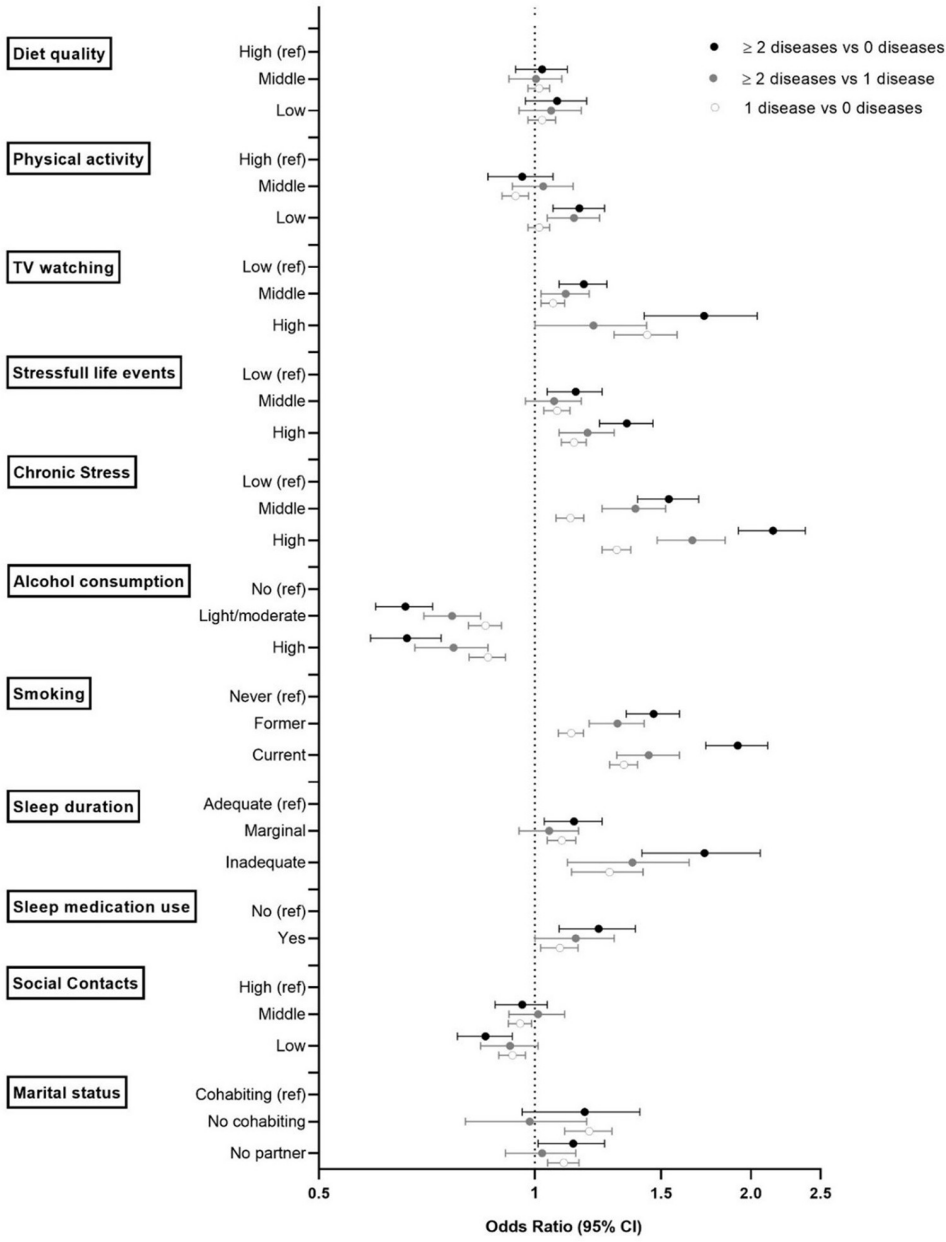
Comparison of lifestyle patterns by number of major chronic diseases. We adjusted multinomial logistic regression models for age, sex, education, household equivalent income and weight status, and mutually adjusted for the lifestyle factors. Data points show the odds ratios with their 95% confidence intervals. The actual numbers are presented in S3 Table in S1 File.

Prevalent multimorbsidity was related with all lifestyle factors, except diet quality (**S3 Table in S1 File and Fig 2**). Patients with multimorbidity had low physical activity levels (< 150 min of MVPA/week) while those with a single disease had not when compared with those with no disease (**S3 Table in S1 File and Fig 2**). Patients with any number of chronic diseases were related with all other lifestyle factors, but the ORs were larger for multimorbidity than for single disease. The highest ORs were found for chronic stress [2.14 (95%CI: 1.92–2.38)], followed by relatively high ORs for smoking [1.91 (95%CI: 1.73–2.11)], inadequate sleep [1.70 (95%CI: 1.41–2.06)] and TV watching [1.70 (95%CI: 1.42–2.04)] (**Fig 2**). Interestingly, when compared with alcohol non-consumers and never smokers, a high alcohol intake was associated with an OR below one and being a former smoker was associated with an OR above one, the magnitudes being greatest in the multimorbidity group [0.66 (95%CI: 0.59–0.74) for high alcohol intake, 1.46 (95%CI: 1.34–1.59) for former smoking]. More complex patterns arose for social relationships. For both multimorbidity and single disease, ORs were below one for low social contact [0.85 (95%CI: 0.78–0.93) and 0.93 (95%CI: 0.89–0.97)] and tended to be above one for having no partner [1.13 (95%CI: 1.01–1.25) and 1.10 (95%CI: 1.04–1.15)]. Associations between lifestyle factors and each chronic disease revealed that more TV watching, less alcohol consumption, any smoking history, more stressful life events and higher chronic stress levels were associated with each disease (**S4 Table in S1 File**). For the other lifestyle factors, we found heterogeneous associations with the different chronic diseases.

## Discussion

The most remarkable findings in this study concerned the associations of higher stress and poor sleep with multimorbidity related to the Big Four chronic diseases. Furthermore, patients with multimorbidity also tended to be physically inactive, contrasting with cases that had a single disease or no major chronic disease. Although smoking history was associated with the presence of multimorbidity and single disease, the associations were stronger for multimorbidity. Of note, alcohol consumption was lower in subjects with multimorbidity and diet quality was not associated with multimorbidity.

### Comparison with existing literature

Existing literature has paid little attention to stress and sleep that people who suffer from multimorbidity may consider highly relevant. Psychosocial stress is associated with chronic disease [43]. A large study of over 13 million subjects convincingly showed that people living with cardiometabolic multimorbidity have an OR of 1.89 (95%CI, 1.49–2.41) for ‘perceived life stress’ compared with those without such conditions [16]. This is comparable with the OR for high chronic stress in relation to multimorbidity in the present study (OR, 2.14; 95%CI, 1.92–2.38). Chronically high stress is an emerging lifestyle-related factor that may significantly reduce quality of life [44]. Additionally, sleep is increasingly recognized as a relevant lifestyle factor in patients with multimorbidity. Data from the US indicates that subjects with inadequate sleep (7–8 hour cut-off) have an OR of 1.8 (95%CI, 1.8–1.9) for multimorbidity [45] and data from China Health indicates an OR of 1.6 (95%CI, 1.4–1.7) for multimorbidity with < 7 hours of sleep [46], although different disease ranges were included to define multimorbidity, which makes the comparison difficult. The OR for inadequate sleep in the present analysis was 1.70 (1.41–2.06) for multimorbidity compared to no disease, and thus similar to previous large cross-sectional studies. In our study, the prevalence of inadequate sleep was high with 22% of patients with multimorbidity reporting inadequate or marginally adequate sleep durations. Thus, chronic stress and poor sleep require serious consideration when designing supportive lifestyle programs for patients with multimorbidity.

Inactivity was specific for prevalent multimorbidity, having no relationship to single disease. In general, many studies have found a relationship between multimorbidity and inactivity [45, 47], although some findings lack significance. [16, 17, 46] and no comparisons against those with a single disease were reported. Compared to the single disease group, we found that multimorbidity appeared more closely related to low physical functioning. This may depend on the definition, with cardiovascular disease and type 2 diabetes tending to be associated with inactivity (S4 Table in S1 File), but it could reflect the accumulation of diseases that lead to physical inability. Irrespective of being a cause or consequence of multimorbidity, inactivity is a strong risk factor for mortality in the ageing process [1], being relevant for both healthy people and patients [3].

It was interesting to find that participants with multimorbidity were more likely to be non-drinkers and former smokers than those with no disease. This may reflect the cross-sectional design, which ignored the temporal relationship between lifestyle behaviours and disease development. For example, while smoking clearly increases the risk of disease [16, 45], having a chronic disease may then prompt lifestyle change such as stop drinking and quit smoking due to the treatment, lifestyle advice or rehabilitation programs. Moreover, despite our finding that people may quit smoking more often after being diagnosed with multiple chronic diseases, there remains a higher risk for multimorbidity. Another study reported a higher odds for multimorbidity among former smokers compared to never smokers [47], with a similar OR (1.36; 95%CI, 1.22–1.52). Nevertheless, many in our participants still smoked (23%), supporting the continued need for smoking cessation programmes.

Consistent with other large-scale studies that only investigated fruit and vegetable intake [16, 17, 45], no association existed between poor diet quality and multimorbidity. This may reflect the association between diet and multimorbidity being too complex to capture with relatively simple indices. More detailed analyses on data-driven dietary patterns from the Lifelines cohort have shown that participants in the highest quintile of a ‘meat, alcohol and potato pattern’ and ‘snack pattern’ had higher odds for multimorbidity, whereas those adhering more to a ‘bread and sweets pattern’ and ‘vegetable, fish and fruit pattern’ had lower odds for multimorbidity [48]. Equally, however, some people may have adjusted their diet upon diagnosis and some may not, obscuring any true association.

Stress management is a prerequisite for successful lifestyle changes in lifestyle intervention programs [49], whereas the social network can serve as a barrier or a facilitator [50]. Unlike stress, however, social connectedness did not stand out as a characteristic typical of multimorbidity. Individuals with at least one chronic disease were more often single but had larger social networks than individuals without chronic disease. Being single may indicate the death of a spouse due to old age or shared unhealthy lifestyles, while the larger social networks may reflect adaptation to health needs. Unfortunately, the current study only included quantitative measures of the social network, without information about the quality of that support, including affection and the absence of loneliness. These factors warrant exploration as underlying reasons for the associations between social relationships and multimorbidity. Improved social resources will help support patients with chronic disease self-management [51].

Multimorbidity may be related to lifestyle through various mechanisms. Many diseases share common pathophysiologies, such as inflammation and accelerated ageing of organ systems [52], and these may be affected by the various lifestyle factors in this analysis. Similarly, the diseases may have unrelated pathophysiologies but may share risk factors (e.g., smoking). Lifestyle and lifestyle-related factors may also reflect social, psychosocial and behavioural elements of health, being associated with both health literacy and self-management skills.

### Strengths and weaknesses

Using data from the Lifelines cohort ensured detailed measurements of a wide range of exposures and clinical outcomes, with the current analysis uniquely combining multiple detailed exposures for all six domains relevant to lifestyle medicine (e.g., many studies have used BMI or weight status in the absence of proper measures of diet intake, but we had access to extensive data on this factor). We also mutually adjusted the lifestyle factors, ensuring better estimates for the independent associations. However, using questionnaires to assess lifestyle factors could have introduced recall bias and socially desirable answers. Our analyses also did not account for family structure because evidence shows that adjustment for familial relatedness only minimally affects the result in current Lifelines samples [53]. Defining multimorbidity based on the Big Four chronic diseases, using both self-report and physical measurements, represents another strength. Previous studies have varied widely in their definitions of multimorbidity, ranging from only including cardiometabolic diseases [16] to including 27 chronic conditions [18], with some potentially less strongly related to lifestyle (e.g. joint pain, frequent headache, allergies). In addition, we included a relatively large number of participants with multimorbidity in a representative population, allowing for meaningful comparisons to the other groups [54].

## Conclusions

Substantial potential exists to improve physical inactivity and smoking cessation and to work on stress management and sleep hygiene interventions when managing patients with multimorbidity. Studies must now determine the relevance of each lifestyle factor on prognosis, should seek to develop lifestyle interventions for patients living with multimorbidity and should investigate the impact of these interventions on quality of life and clinical outcomes.

## Supporting information

S1 File (DOCX)
