# Supplementary material for: Lifestyle factors related to prevalent chronic disease multimorbidity: A population-based cross-sectional study
Source: PLoS One. 2023 Jul 24;18(7):e0287263. doi: 10.1371/journal.pone.0287263 (PMC10365307; doi:10.1371/journal.pone.0287263)
Supplement: S1 File — (DOCX) [file pone.0287263.s001.docx]

**Supplemental Table 1 Comparison of baseline characteristics of the excluded sample with the included (analytic) sample**

|  | | **Excluded (N=73,383)** | **Included (N=79,345)** | **P-value** |
| --- | --- | --- | --- | --- |
| **General characteristics** | |  |  |  |
| Mean age (SD); range | | 44.9 (13.8);18-93 | 44.4 (12.5);18-90 | <0.001 |
| Gender (Male) (%) | | 42.5 | 40.6 | <0.001 |
| Educational Attainment (%) | |  |  | <0.001 |
|  | Elementary | 3.9 | 2.0 |  |
|  | Lower secondary | 29.6 | 25.8 |  |
|  | Upper secondary | 38.9 | 40.1 |  |
|  | Tertiary | 27.5 | 32.1 |  |
| Household Equivalent Income (€/month) (%) | |  |  | <0.001 |
|  | <1100 | 19.9 | 16.6 |  |
|  | 1100–1499 | 22.6 | 22.6 |  |
|  | 1500–1899 | 24.0 | 26.6 |  |
|  | ≥1900 | 18.1 | 20.4 |  |
|  | Don’t know/don’t tell | 15.4 | 13.8 |  |
| Mean BMI (SD); range | | 26.1 (4.5);13.4-73.6 | 26.0 (4.2);15.2-58.6 | <0.001 |
| Weight status2 (%) | |  |  | <0.001 |
|  | Underweight | 0.9 | 0.7 |  |
|  | Normal weight | 43.5 | 44.4 |  |
|  | Overweight | 39.3 | 39.6 |  |
|  | Obesity | 16.4 | 15.2 |  |
| **Chronic diseases** | |  |  |  |
| CVD total (%) | | 11.3 | 10.9 | 0.009 |
| CVD by type (%)3 | |  |  |  |
|  | Self-reported heart valve problems | 9.3 | 9.3 | 0.862 |
|  | Self-reported atherosclerosis | 4.2 | 4.1 | 0.762 |
|  | Self-reported thrombosis | 10.6 | 11.5 | 0.046 |
|  | Myocardial infarct | 10.2 | 7.2 | <0.001 |
|  | Heart failure | 5.1 | 4.4 | 0.018 |
|  | Self-reported CABG | 15.2 | 11.3 | <0.001 |
|  | Self-reported aneurysm | 3.4 | 2.1 | <0.001 |
|  | Self-reported stroke | 8.5 | 5.5 | <0.001 |
|  | Self-reported narrowing carotid | 2.4 | 2.2 | 0.402 |
|  | Atrial fibrillation | 5.7 | 4.2 | <0.001 |
|  | Self-reported arrhythmia | 61.7 | 69.0 | <0.001 |
| Cancer total (%) | | 2.7 | 2.3 | <0.001 |
| Cancer by type (%)4 | |  |  | 0.222 |
|  | Solid tumors | 88.0 | 87.8 |  |
|  | Bone and soft tissue cancer | 1.5 | 2.4 |  |
|  | Hematological cancer | 8.4 | 8.0 |  |
|  | Central nervous system cancer | 2.1 | 1.8 |  |
| Respiratory disease total (%) | | 22.1 | 22.2 | 0.930 |
| Respiratory disease by type (%)5 | |  |  |  |
|  | Asthma | 58.2 | 57.1 | 0.125 |
|  | Chronic airway obstruction | 64.2 | 64.6 | 0.549 |
| Diabetes Type 2 | | 3.5 | 2.7 | <0.001 |

**Supplemental Table 1 (Continued)**

| **Lifestyle factors** | | **Excluded (N=73,383)** | **Included (N=79,345)** | **P-value** |
| --- | --- | --- | --- | --- |
| **Nutrition** | |  |  | 0.001 |
|  | High (T3, score 27-44) | 33.6 | 34.3 |  |
|  | Middle (T2, score 22-26) | 36.7 | 37.0 |  |
|  | Low (T1, score 1-21) | 29.6 | 28.7 |  |
| **Exercise** | |  |  |  |
| Physical activity | |  |  | 0.270 |
|  | High (≥300 min/week) | 35.0 | 34.7 |  |
|  | Middle (150-299 min/week) | 24.3 | 24.7 |  |
|  | Low (˂150 min/week) | 40.6 | 40.7 |  |
| TV watching | |  |  | <0.001 |
|  | Low (≤2 hrs/day) | 52.2 | 51.5 |  |
|  | Middle (3-4 hrs/day) | 44.9 | 46.1 |  |
|  | High (≥5 hrs/day) | 2.9 | 2.4 |  |
| **Stress** | |  |  |  |
| Stressful life events (%) | |  |  | <0.001 |
|  | Low (0 events) | 43.0 | 44.2 |  |
|  | Middle (1 event) | 27.0 | 27.4 |  |
|  | High (≥2 events) | 30.0 | 28.5 |  |
| Chronic stress (%) | |  |  |  |
|  | Low (0 factors) | 23.0 | 21.5 | <0.001 |
|  | Middle (1 or 2 factors) | 37.3 | 37.9 |  |
|  | High (≥3 factors) | 39.7 | 40.6 |  |
| **Substance abuse** | |  |  | <0.001 |
| Alcohol consumption (%) | |  |  |  |
|  | No | 27.4 | 16.8 |  |
|  | Light/moderate | 51.5 | 61.0 |  |
|  | High | 21.1 | 22.3 |  |
| Smoking habits (%) | |  |  | <0.001 |
|  | Never smoker | 45.5 | 46.4 |  |
|  | Former smoker | 32.9 | 33.0 |  |
|  | Current smoker | 21.7 | 20.6 |  |
| **Sleep** | |  |  |  |
| Sleep duration (%) | |  |  | <0.001 |
|  | Adequate | 82.5 | 84.5 |  |
|  | Marginally (too short/long) | 15.2 | 13.7 |  |
|  | Inadequate (too short/long) | 2.3 | 1.8 |  |
| Sleep medication use | |  |  |  |
|  | Yes | 8.1 | 7.7 | 0.001 |
| **Relationships** | |  |  |  |
| Number of contacts (%) | |  |  | <0.001 |
|  | High (≥20) | 38.9 | 35.5 |  |
|  | Middle (10-19) | 33.3 | 35.0 |  |
|  | Low (≤9) | 27.8 | 29.5 |  |
| Marital Status (%) | |  |  | <0.001 |
|  | Relationship with cohabiting | 78.8 | 81.1 |  |
|  | Relationship with no cohabiting | 5.4 | 5.2 |  |
|  | No partner | 15.7 | 13.7 |  |

2 Underweight is BMI˂18.5, Normal weight is 18.5≤BMI˂25.0, Overweight is 25≤BMI˂30.0 and Obesity is BMI≥30.0

3 Percentages CVD by type are the percentages within the group of respondents with prevalent CVD

4 Percentages cancer by type are the percentages within the group of respondents with prevalent cancer. The types of cancer represent primary types, therefore categories are mutually exclusive.

5 Percentages respiratory disease by type are the percentages within the group of respondents with prevalent respiratory disease

The difference for age and BMI between included and excluded samples was tested by independent t test, the difference for all the other variables was tested by chi-square test.

**Supplemental Table 2 General characteristics and lifestyle profiles of participants with chronic disease**

|  | | CVD | Cancer | Respiratory Disease | T2D |
| --- | --- | --- | --- | --- | --- |
| N | | 8,638 | 1,848 | 17,594 | 2,169 |
| **General characteristics** | |  |  |  |  |
| Mean age (SD);  Range | | 51.8 (12.6);  18-88 | 54.7 (12.1);  18-87 | 44.0 (12.7);  18-90 | 56.8 (10.7);  25-88 |
| Gender (Male) (%) | | 41.6 | 31.0 | 40.2 | 50.5 |
| Educational Attainment (%) | |  |  |  |  |
|  | Elementary | 3.8 | 3.5 | 2.7 | 7.2 |
|  | Lower secondary | 34.2 | 38.1 | 27.7 | 42.1 |
|  | Upper secondary | 35.7 | 32.0 | 40.2 | 31.8 |
|  | Tertiary | 26.3 | 26.4 | 29.4 | 18.9 |
| Household Equivalent Income (in € per month) (%) | |  |  |  |  |
|  | <1100 | 15.6 | 14.5 | 18.6 | 15.8 |
|  | 1100–1499 | 22.4 | 20.3 | 22.8 | 24.1 |
|  | 1500–1899 | 24.0 | 23.5 | 25.7 | 21.3 |
|  | ≥1900 | 22.7 | 26.3 | 19.0 | 22.3 |
|  | Don’t know/don’t tell | 15.2 | 15.4 | 13.9 | 16.6 |
| Mean BMI (SD);  Range | | 26.9 (4.4);  16.8-54.5 | 26.8 (4.5);  17.3-54.6 | 26.2 (4.5);  15.2-55.9 | 30.3 (5.1);  17.9-51.4 |
| Weight status2 (%) | |  |  |  |  |
|  | Underweight | 0.4 | 0.6 | 0.7 | 0.1 |
|  | Normal weight | 35.8 | 36.9 | 43.3 | 12.1 |
|  | Overweight | 43.8 | 43.6 | 39.3 | 40.9 |
|  | Obesity | 20.5 | 18.9 | 16.7 | 46.9 |
| **Lifestyle factors** | |  |  |  |  |
| Nutrition | |  |  |  |  |
| Diet quality (LLDS) (%) | |  |  |  |  |
|  | High (T3, score 27-44) | 40.2 | 45.0 | 31.8 | 40.5 |
|  | Middle (T2, score 22-26) | 35.7 | 34.3 | 37.3 | 37.2 |
|  | Low (T1, score 1-21) | 24.1 | 20.7 | 30.9 | 22.3 |
| Exercise | |  |  |  |  |
| Physical activity | |  |  |  |  |
|  | High (≥300 min/week) | 34.3 | 37.9 | 34.1 | 32.7 |
|  | Middle (150-299 min/week) | 22.9 | 23.2 | 23.2 | 20.4 |
|  | Low (˂150 min/week) | 42.7 | 39.0 | 42.7 | 46.8 |
| TV watching (hrs/day) (%) | |  |  |  |  |
|  | Low (≤2 hrs/day) | 44.6 | 41.4 | 49.1 | 33.7 |
|  | Middle (3-4 hrs/day) | 51.5 | 54.5 | 47.7 | 59.0 |
|  | High (≥5 hrs/day) | 3.9 | 4.1 | 3.2 | 7.3 |
| Stress | |  |  |  |  |
| Stressful life event (%) | |  |  |  |  |
|  | Low (0 events) | 38.2 | 37.9 | 40.9 | 38.2 |
|  | Middle (1 event) | 28.2 | 27.5 | 27.3 | 37.2 |
|  | High (≥2 events) | 33.5 | 34.6 | 31.7 | 34.7 |
| Chronic stress (%) | |  |  |  |  |
|  | Low (0 factors) | 22.4 | 26.2 | 18.6 | 30.1 |
|  | Middle (1 or 2 factors) | 37.6 | 38.3 | 36.2 | 38.9 |
|  | High (≥3 factors) | 39.9 | 35.4 | 45.1 | 31.1 |
| Substance abuse | |  |  |  |  |
| Alcohol consumption (%) | |  |  |  |  |
|  | No | 20.7 | 21.4 | 17.8 | 28.9 |
|  | Light/moderate | 58.4 | 59.4 | 58.2 | 52.6 |
|  | High | 20.9 | 19.2 | 24.1 | 18.5 |
| Smoking habits (%) | |  |  |  |  |
|  | Never smoker | 37.4 | 37.2 | 41.3 | 32.7 |
|  | Former smoker | 43.8 | 45.5 | 32.2 | 49.9 |
|  | Current smoker | 18.8 | 17.4 | 26.5 | 17.4 |
| Sleep | |  |  |  |  |
| Sleep duration (%) | |  |  |  |  |
|  | Adequate | 79.8 | 80.4 | 82.8 | 76.8 |
|  | Marginal (too short/long) | 16.9 | 17.1 | 14.9 | 18.6 |
|  | Inadequate (too short/long) | 3.2 | 2.5 | 2.3 | 4.6 |
| Sleep medication use | |  |  |  |  |
|  | Yes | 9.8 | 9.6 | 8.4 | 6.1 |
| Relationships | |  |  |  |  |
| Number of contacts (%) | |  |  |  |  |
|  | High (≥20) | 35.2 | 36.0 | 36.4 | 36.8 |
|  | Middle ( 10-19) | 34.4 | 36.9 | 34.5 | 31.1 |
|  | Low (≤9) | 30.4 | 27.1 | 29.1 | 32.1 |
| Marital Status (%) | |  |  |  |  |
|  | Relationship with cohabiting | 83.6 | 83.3 | 79.0 | 82.4 |
|  | Relationship with no cohabiting | 3.8 | 2.7 | 5.9 | 1.9 |
|  | No partner | 12.6 | 14.0 | 15.1 | 15.7 |

Underweight is BMI˂18.5, Normal weight is 18.5≤BMI˂25.0, Overweight is 25≤BMI˂30.0 and Obesity is BMI≥30.0 Patients with more than one disease were not excluded from the analysis.

**Supplemental Table 3.** **Lifestyle factors in relation to having multimorbidity as compared to having one of no major chronic disease**

|  | Number of diseases | | |
| --- | --- | --- | --- |
|  | *≥2 (vs. 0)* | *≥2 (vs. 1)* | *1 (vs. 0)* |
| **Nutrition** |  |  |  |
| Diet Quality (LLDS) |  |  |  |
| High (T3, score 27-44) | Ref. | Ref. | Ref. |
| Middle (T2, score 22-26) | 1.02 (0.94 – 1.11) | 1.00 (0.92 – 1.09) | 1.01 (0.98 – 1.05) |
| Low (T1, score 1-21) | 1.07 (0.97 – 1.18) | 1.05 (0.95 – 1.16) | 1.02 (0.98 – 1.07) |
| **Exercise** |  |  |  |
| Physical activity |  |  |  |
| High (≥300 min/week) | Ref. | Ref. | Ref. |
| Middle (150-299 min/week) | 0.96 (0.88 – 1.06) | 1.02 (0.93 – 1.13) | **0.94 (0.90 – 0.98)** |
| Low (˂150 min/week) | **1.15 (1.06 – 1.25)** | **1.13 (1.04 – 1.23)** | 1.01 (0.98 – 1.05) |
| TV watching |  |  |  |
| Low (≤2 hrs/day) | Ref. | Ref. | Ref. |
| Middle (3-4 hrs/day) | **1.17 (1.08 – 1.26)** | **1.10 (1.02 – 1.19)** | **1.06 (1.02 – 1.10)** |
| High (≥5 hrs/day) | **1.70 (1.42 – 2.04)** | 1.19 (1.00 – 1.43) | **1.43 (1.29 – 1.58)** |
| **Stress** |  |  |  |
| Stressful life events |  |  |  |
| Low (0 events) | Ref. | Ref. | Ref. |
| Middle (1 event) | **1.14 (1.04 – 1.24)** | 1.06 (0.97 – 1.16) | **1.07 (1.03 – 1.12)** |
| High (≥2 events) | **1.34 (1.23 – 1.46)** | **1.18 (1.08 – 1.29)** | **1.13 (1.09 – 1.18)** |
| Chronic stress |  |  |  |
| Low (0 factors) | Ref. | Ref. | Ref. |
| Middle (1 or 2 factors) | **1.53 (1.39 – 1.69)** | **1.38 (1.24 – 1.52)** | **1.12 (1.07 – 1.17)** |
| High (≥3 factors) | **2.14 (1.92 – 2.38)** | **1.65 (1.48 – 1.84)** | **1.30 (1.24 – 1.36)** |
| **Substance abuse** |  |  |  |
| Alcohol consumption |  |  |  |
| No | Ref. | Ref. | Ref. |
| Light /moderate | **0.66 (0.60 – 0.72)** | **0.76 (0.70 – 0.84)** | **0.85 (0.81 – 0.90)** |
| High | **0.66 (0.59 – 0.74)** | **0.77 (0.68 – 0.86)** | **0.86 (0.81 – 0.91)** |
| Smoking |  |  |  |
| Never smoker | Ref. | Ref. | Ref. |
| Former smoker | **1.46 (1.34 – 1.59)** | **1.30 (1.19 – 1.42)** | **1.12 (1.08 – 1.17)** |
| Current smoker | **1.91 (1.73 – 2.11)** | **1.43 (1.30 – 1.59)** | **1.33 (1.27 – 1.39)** |
| **Sleep** |  |  |  |
| Sleep Duration |  |  |  |
| Adequate | Ref. | Ref. | Ref. |
| Marginal (too short/long) | **1.13 (1.03 – 1.24)** | 1.04 (0.95 – 1.15) | **1.09 (1.04 – 1.14)** |
| Inadequate (too short/long) | **1.70 (1.41 – 2.06)** | **1.35 (1.11 – 1.64)** | **1.26 (1.13 – 1.42)** |
| Sleep medication use |  |  |  |
| No | Ref. | Ref. | Ref. |
| Yes | **1.22 (1.08 – 1.38)** | 1.13 (1.00 – 1.29) | **1.08 (1.02 – 1.15)** |
| **Relationships** |  |  |  |
| Number of contacts |  |  |  |
| High (≥20) | Ref. | Ref. | Ref. |
| Middle ( 10-19) | 0.96 (0.88 – 1.04) | 1.01 (0.92 – 1.10) | **0.95 (0.92 – 0.99)** |
| Low (≤9) | **0.85 (0.78 – 0.93)** | 0.92 (0.84 – 1.01) | **0.93 (0.89 – 0.97)** |
| Marital status |  |  |  |
| Relationship with cohabiting | Ref. | Ref. | Ref. |
| Relationship with no cohabiting | 1.16 (0.96 – 1.40) | 0.97 (0.80 – 1.18) | **1.19 (1.10 – 1.28)** |
| No partner | **1.13 (1.01 – 1.25)** | 1.02 (0.91 – 1.14) | **1.10 (1.04 – 1.15)** |

Data are presented as Odds Ratio (95% confidence interval). Multinomial logistic regression model on multimorbidity compared to having no chronic disease (≥2 vs. 0) or to having a single chronic disease (≥2 vs. 1). For completeness, also results of having a single chronic disease compared to having no chronic disease (1 vs. 0) are presented. Models are adjusted for age, sex, education, household equivalent income and weight status.

**Supplemental Table 4. Lifestyle factors in relation to having a specific major chronic disease**

|  | CVD | Cancer | Respiratory Disease | T2D |
| --- | --- | --- | --- | --- |
| **Nutrition** |  |  |  |  |
| Diet Quality (LLDS) |  |  |  |  |
| High (T3, score 27-44) | Ref. | Ref. | Ref. | Ref. |
| Middle (T2, score 22-26) | 0.95 (0.90 – 1.00) | 0.96 (0.86 – 1.08) | **1.07 (1.02 – 1.11)** | 1.02 (0.91 – 1.13) |
| Low (T1, score 1-21) | 0.97 (0.90 – 1.03) | 1.00 (0.88 – 1.15) | **1.08 (1.03 – 1.14)** | 0.89 (0.78 – 1.02) |
| **Exercise** |  |  |  |  |
| Physical activity |  |  |  |  |
| High (≥300 min/week) | Ref. | Ref. | Ref. | Ref. |
| Middle (150-299 min/week) | 1.00 (0.94 – 1.06) | 0.98 (0.87 – 1.12) | **0.94 (0.90 – 0.99)** | 1.01 (0.89 – 1.15) |
| Low (˂150 min/week) | **1.11 (1.05 – 1.18)** | 0.97 (0.87 – 1.09) | 1.02 (0.98 – 1.06) | **1.20 (1.08 – 1.33)** |
| TV watching (hrs/day) |  |  |  |  |
| Low (≤2 hrs/day) | Ref. | Ref. | Ref. | Ref. |
| Middle (3-4 hrs/day) | 1.04 (0.99 – 1.10) | **1.11 (1.00 – 1.23)** | **1.07 (1.03 – 1.11)** | **1.16 (1.04 – 1.28)** |
| High (≥5 hrs/day) | **1.29 (1.13 – 1.48)** | **1.35 (1.04 – 1.75)** | **1.32 (1.19 – 1.47)** | **1.76 (1.44 – 2.16)** |
| **Stress** |  |  |  |  |
| Stressful life events |  |  |  |  |
| Low (0 events) | Ref. | Ref. | Ref. | Ref. |
| Middle (1 event) | **1.11 (1.05 – 1.18)** | 1.07 (0.95 – 1.21) | **1.05 (1.01 – 1.10)** | 1.02 (0.91 – 1.15) |
| High (≥2 events) | **1.21 (1.14 – 1.28)** | **1.22 (1.08 – 1.37)** | **1.12 (1.07 – 1.16)** | **1.13 (1.01 – 1.26)** |
| Chronic stress |  |  |  |  |
| Low (0 factors) | Ref. | Ref. | Ref. | Ref. |
| Middle (1 or 2 factors) | **1.30 (1.22 – 1.39)** | **1.22 (1.08 – 1.38)** | **1.14 (1.09 – 1.20)** | **1.19 (1.06 – 1.33)** |
| High (≥3 factors) | **1.64 (1.53 – 1.75)** | **1.48 (1.28 – 1.70)** | **1.31 (1.25 – 1.38)** | **1.29 (1.13 – 1.48)** |
| **Substance abuse** |  |  |  |  |
| Alcohol consumption |  |  |  |  |
| No | Ref. | Ref. | Ref. | Ref. |
| Light /moderate | **0.79 (0.74 – 0.85)** | **0.84 (0.74 – 0.95)** | **0.90 (0.86 – 0.94)** | **0.55 (0.49 – 0.62)** |
| High | **0.76 (0.70 – 0.82)** | **0.80 (0.68 – 0.93)** | **0.94 (0.89 – 1.00)** | **0.46 (0.40 – 0.54)** |
| Smoking |  |  |  |  |
| Never smoker | Ref. | Ref. | Ref. | Ref. |
| Former smoker | **1.21 (1.15 – 1.28)** | **1.15 (1.03 – 1.29)** | **1.13 (1.08 – 1.17)** | **1.32 (1.18 – 1.47)** |
| Current smoker | **1.18 (1.10 – 1.26)** | **1.21 (1.05 – 1.40)** | **1.49 (1.43 – 1.56)** | **1.39 (1.21 – 1.60)** |
| **Sleep** |  |  |  |  |
| Sleep Duration |  |  |  |  |
| Adequate | Ref. | Ref. | Ref. | Ref. |
| Marginally (too short/long) | **1.08 (1.01 – 1.15)** | 1.08 (0.94 – 1.22) | **1.07 (1.02 – 1.13)** | 1.04 (0.92 – 1.18) |
| Inadequate (too short/long) | **1.38 (1.20 – 1.60)** | 1.01 (0.74 – 1.37) | **1.23 (1.09 – 1.39)** | **1.63 (1.29 – 2.05)** |
| Sleep medication use |  |  |  |  |
| Yes | **1.27 (1.17 – 1.38)** | 1.10 (0.93 – 1.30) | 1.03 (0.97 – 1.10) | **0.82 (0.68 – 0.99)** |
| **Relationships** |  |  |  |  |
| Number of contacts |  |  |  |  |
| High (≥20) | Ref. | Ref. | Ref. | Ref. |
| Middle ( 10-19) | 1.01 (0.96 – 1.07) | 1.03 (0.92 – 1.15) | **0.94 (0.91 – 0.98)** | 0.93 (0.84 – 1.05) |
| Low (≤9) | 0.98 (0.92 – 1.04) | 0.82 (0.72 – 0.92) | **0.92 (0.88 – 0.96)** | 1.01 (0.90 – 1.12) |
| Marital status |  |  |  |  |
| Relationship with cohabiting | Ref. | Ref. | Ref. | Ref. |
| Relationship with no cohabiting | **1.14 (1.00 – 1.28)** | 0.90 (0.67 – 1.21) | **1.10 (1.02 – 1.19)** | **0.83 (0.60 – 1.15)** |
| No partner | 0.96 (0.89 – 1.04) | 1.04 (0.90 – 1.20) | **1.06 (1.01 – 1.11)** | **1.29 (1.13 – 1.48)** |

Binary logistics regression analyses on Cardiovascular Disease (CVD), Cancer, Respiratory Disease and Diabetes Type 2 (T2D). The models are fully adjusted and odds ratio’s and 95% confidence intervals are presented. Patients with more than one chronic condition were not excluded from the analysis.
